# The Long-Term Effectiveness of Omalizumab in Adult Patients with Severe Allergic Asthma: Continuous Treatment Versus Boosting Treatment

**DOI:** 10.3390/jcm10040707

**Published:** 2021-02-11

**Authors:** Wei-Chang Huang, Pin-Kuei Fu, Ming-Cheng Chan, Chun-Shih Chin, Wen-Nan Huang, Kuo-Lung Lai, Jiun-Long Wang, Wei-Ting Hung, Yi-Da Wu, Chia-Wei Hsieh, Ming-Feng Wu, Yi-Hsing Chen, Jeng-Yuan Hsu

**Affiliations:** 1 Department of Internal Medicine, Division of Chest Medicine, Taichung Veterans General Hospital, Taichung 407, Taiwan; huangweichangtw@gmail.com (W.-C.H.); drchan@vghtc.gov.tw (M.-C.C.); chungshih@vghtc.gov.tw (C.-S.C.); wangjilong1217@gmail.com (J.-L.W.); osmigo@seed.net.tw (M.-F.W.); 2 Department of Medical Technology, Jen-Teh Junior College of Medicine, Nursing and Management, Miaoli 350, Taiwan; 3 Ph.D. Program in Translational Medicine, National Chung Hsing University, Taichung 402, Taiwan; chiaweih@gmail.com; 4 School of Medicine, Chung Shan Medical University, Taichung 402, Taiwan; 5 Department of Industrial Engineering and Enterprise Information, Tunghai University, Taichung 407, Taiwan; 6 Department of Critical Care Medicine, Taichung Veterans General Hospital, Taichung 407, Taiwan; yetquen@gmail.com; 7 College of Human Science and Social Innovation, Hungkuang University, Taichung 433, Taiwan; 8 Department of Computer Science, Tunghai University, Taichung 407, Taiwan; 9 Department of Internal Medicine, Division of Critical Care and Respiratory Therapy, Taichung Veterans General Hospital, Taichung 407, Taiwan; 10 Central Taiwan University of Science and Technology, Taichung 406, Taiwan; 11 The College of Science, Tunghai University, Taichung 407, Taiwan; 12 Immunology and Rheumatology, Department of Internal Medicine, Division of Allergy, Taichung Veterans General Hospital, Taichung 407, Taiwan; gtim5555@yahoo.com (W.-N.H.); kllaichiayi@yahoo.com.tw (K.-L.L.); wtinghung@gmail.com (W.-T.H.); bagidr1@hotmail.com (Y.-D.W.); 13 Agricultural Biotechnology Research Center, National Chung Hsing University, Taichung 402, Taiwan; 14 Department of Life Sciences, National Chung Hsing University, Taichung 402, Taiwan; 15 Department of Medical Laboratory Science and Biotechnology, Central Taiwan University of Science and Technology, Taichung 406, Taiwan; 16 Faculty of Medicine, National Yang-Ming University, Taipei 112, Taiwan; 17 Department of Medical Research, Division of Clinical Research, Taichung Veterans General Hospital, Taichung 407, Taiwan; 18 School of Medicine, China Medical University, Taichung 404, Taiwan; 19 School of Physical Therapy, Chung-Shan Medical University, Taichung 402, Taiwan

**Keywords:** boost, long-term effectiveness, Omalizumab, real-world, severe allergic asthma

## Abstract

The implications of boosting Omalizumab treatment (OT) in patients with severe allergic asthma (SAA) remain unclear. The study aimed to explore and compare the 12-month effectiveness between continuous, at least 10-month OT (continuation group) and four-month boost of Omalizumab (boost group) in adult patients with SAA. In this retrospective cohort study, clinical data were collected for further analysis. Of all participants (*n* = 124), a significant reduction in annual exacerbations (baseline = 0.8 ± 1.5, follow-up = 0.5 ± 1.0, *p* = 0.047 *) and improvement in small airway ventilation as evaluated by forced expiratory flow at 25–75% (baseline = 55.1 ± 11.1%, follow-up = 59.4 ± 8.4%, *p* < 0.001 *) were found in the continuation group (*n* = 110). By contrast, the boost group (*n* = 14) had significantly increased annual exacerbations (baseline = 0.7 ± 1.4, follow-up = 2.9 ± 3.6, *p* = 0.031 *) and impaired small airway function (baseline = 55.3 ± 12.9, follow-up = 52.1 ± 12.5, *p* = 0.026 *). Furthermore, the continuation group rather than the boost group had significant decreases in the frequency of oral corticosteroid (OCS) use as controllers (baseline = 32.7%, follow-up = 20.0%, *p* = 0.047 *; baseline = 50.0%, follow-up = 21.4%, *p* = 0.237, respectively) and OCS maintenance dose (mg/month) (baseline = 85.9 ± 180.8, follow-up = 45.8 ± 106.6, *p* = 0.020 *; baseline = 171.4 ± 221.5, follow-up = 50.0 ± 104.3, *p* = 0.064, respectively), and increases in asthma control test scores (baseline = 16.0 ± 3.0, follow-up = 19.8 ± 4.4, *p* < 0.001 *; baseline = 14.6 ± 3.8, follow-up = 19.7 ± 4.7, *p* = 0.050, respectively). Continuous OT would be beneficial for adult patients with SAA, while boost of Omalizumab would worsen their long-term outcomes.

## 1. Introduction

Although asthma is characterized by a collection of respiratory symptoms, including chronic cough, shortness of breath, wheeze and chest tightness, that vary over time and in intensity, it also involves chronic airway inflammation and variable airflow limitation, and is considered a heterogeneous pulmonary disease that involves complex pathophysiologic mechanisms [1].

The severity of asthma is divided into five Global Initiative for Asthma (GINA) steps, ranging from Step 1 to Step 5 and increasing with the treatment intensity required to control symptoms and exacerbations [1]. Severe asthma, defined as asthma that was uncontrolled despite Step 4 or 5 medications, good adherence and inhaler techniques, and maximally optimal treatment of contributory factors [2], has a substantial impact on the patient’s quality of life, as well as medical resource utilization and expenditure even though it only affects a small proportion of patients with asthma [3]. For this reason, the GINA strategy has suggested oral corticosteroids (OCSs), tiotropium bromide, anti-immunoglobulin E (anti-IgE) monoclonal antibodies, anti-interleukin-5 (anti-IL-5) or its receptor alpha subunit monoclonal antibodies and anti-IL-4 receptor monoclonal antibodies as add-on therapies to try and control symptoms and exacerbations in patients with severe asthma [1].

Plenty of real-world studies have shown that long-term, continuous use of Omalizumab, an anti-IgE antibody, can greatly reduce exacerbations, asthma related emergency visits and hospital admissions, OCS maintenance doses and fraction exhaled nitric oxide levels, as well as improve asthma control, lung function and quality of life in adult and pediatric patients with severe allergic asthma (SAA) [4,5,6,7,8,9,10,11,12,13,14]. However, little is known about the impact of short-term boost of Omalizumab on the long-term clinical outcomes in such populations. In addition, there are currently no set recommendations on how long a patient with SAA should be on the treatment of Omalizumab, which factor is able to predict which SAA patients would tolerate withdrawal of Omaluzumab treatment, and which is the optimal protocol to step-down Omalizumab for patients with SAA.

We hypothesized that, with regards to long-term effectiveness, patients with SAA would benefit from continuous use of Omalizumab while boost of Omalizumab would worsen the long-term clinical outcomes in this population. Therefore, the current study aimed to evaluate and compare the long-term, 12-month effectiveness in terms of exacerbation frequency, use of OCS as controllers, OCS maintenance dose, asthma control, pulmonary function measurements and inhaled maintenance pharmacological therapies between continuous Omalizumab therapy for at least 10 months in the follow-up period and boost of Omalizumab treatment for the first four months of the follow-up period in adult patients with SAA.

## 2. Materials and Methods

### 2.1. Study Design, Setting and Population

This retrospective cohort study with a longitudinal comparison (without a matched cohort for comparison) was conducted in accordance with the Declaration of Helsinki. It took place at Taichung Veterans General Hospital (TCVGH), a tertiary teaching hospital in central Taiwan, from January 2010 to January 2019 and was approved by the Institutional Review Board and Ethics Committee of TCVGH (approval no. CE19015B). The need for informed consent from participants was waived because the study was based on a retrospective electronic medical chart review. Patients who were aged 20 years and more, had SAA as per the physician diagnosis and addressed the application for the reimbursement of Omalizumab treatment from Taiwan National Health Insurance (NHI) based on the physician recommendation were enrolled in this study. Subjects were excluded if the reimbursement of Omalizumab treatment was not approved by Taiwan NHI.

### 2.2. Data Collection

The participating physicians reviewed and collected data from the patient’s electronic medical records, and completed a patient record form for each participant, which included their demographic information, smoking history, history of asthma treatment at the study institute, results of multiple allergens simultaneous test, co-morbidities of interest, white blood cell counts, peripheral blood eosinophil percentages and counts, and total IgE levels within 3 months prior to their enrollment. Furthermore, asthma control test (ACT) scores and spirometric data interpreted according to the American Thoracic Society statement within 3 months prior to (baseline) and 12 months after enrollment [15], one-year exacerbation history before (baseline) and after enrollment, and oral and inhaled maintenance pharmacological therapies for asthma at enrollment and 12 months after enrollment were also recorded. Co-morbidities of interest included depression, insomnia, osteoporosis, cerebrovascular disorders (i.e., intracranial hemorrhage and ischemic stroke), gastroesophageal reflux disease, chronic obstructive pulmonary disease, diabetes mellitus, and atopic diseases (i.e., allergic rhinitis, atopic dermatitis, urticaria and allergic conjunctivitis), food or drug allergy, aspirin-exacerbated respiratory disease, and obstructive sleep apnea syndrome. All patient information was anonymized and de-identified prior to analysis. All available data were analyzed after allowing for missing information.

### 2.3. Study Outcomes and Group Definitions

The outcomes of interest in the current study included changes in asthma control as evaluated by ACT scores, frequency of OCS use as a controller, OCS maintenance dose, inhaled maintenance pharmacological therapy, exacerbation frequency and lung function measurement between the enrollment/baseline and 12-month follow-up.

A prescription of OCS for >7 days in the outpatient department was considered as the maintenance pharmacological therapy for severe asthma. An exacerbation was defined as a worsening of symptoms and lung functions that required OCS use for at least 3 days at the outpatient service or emergency visits and even hospital admissions.

Although favorable treatment response observed at four-month after the approval of Omalizumab in all subjects, whether continuation or discontinuation/stepping-down of Omalizumab treatment depended on the judgment and suggestion of the SAA committee members of Taiwan NHI after reviewing the medical records. Based on the committee’s recommendation, participants who received Omalizumab continuously for at least 10 months in the follow-up period were categorized as the continuation group. Those with boost of Omalizumab treatment for four months after its approval for reimbursement from the Taiwan NHI, were considered as the boost group.

### 2.4. Statistical Analysis

Continuous variables were described using the number of observation (percentage), mean and standard deviation (SD), and median and interquartile range. They were compared using a paired sample t-test or Wilcoxon signed-rank test based on the normality assumption between the enrollment/baseline and 12-month follow-up. Categorical variables were tabulated as frequency and percentage and were compared using the Chi-squared test between the baseline and one-year follow-up period. A significant difference was defined by a two-sided *p*-value < 0.05.

## 3. Results

Figure S1 shows that, of the 128 SAA adult patients who applied for Omalizumab reimbursement from the Taiwan NHI during the study period, 124 received approval while four did not. Therefore, a total of 124 subjects (total group), including 110 patients who received Omalizumab treatment for at least 10 months (continuation group) and 14 participants who discontinued their use of Omalizumab after four-months treatment (boost group) because of administrative issues from the Taiwan NHI, were included in the final analysis.

Table 1 presents the baseline demographic and clinical data of all participants. The overall mean age was 60.8 ± 15.7 years while more than half of all the participants were male (67/124, 54.0%) and had never smoked (82/124, 66.1%).

**Figure 1:**
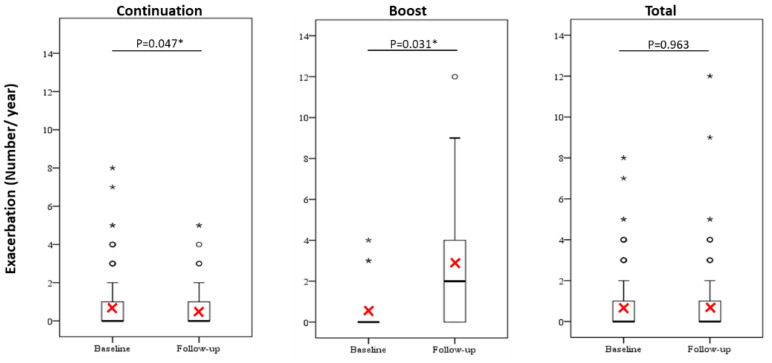
Exacerbation frequency at baseline and at 12-months follow-up. Red cross represents the mean value. The difference was compared using a paired sample *t*-test or Wilcoxon signed-rank test. *p* < 0.05 *.

Exacerbations in the continuation group and boost group were significantly reduced and increased, respectively, during the 12-month follow-up compared with the year prior to enrollment, although it showed no significant change in the total group (Figure 1). Moreover, a higher percentage of participants in the continuation group had complete prevention from (16.4% and 7.1% in the continuation group and boost group, respectively) and had never been present of exacerbations (55.5% and 28.6% in the continuation group and boost group, respectively) (Table S1). Meanwhile, a lower proportion of patients who had continuous Omalizumab treatment in the follow-up period experienced the occurrence of exacerbations when compared to those in the boost group (15.5% and 50.0% in the continuation group and boost group, respectively) (Table S1).

**Figure 2:**
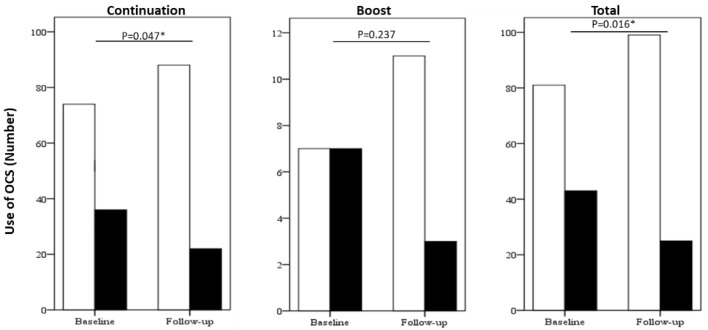
Frequency of OCS use as controllers at baseline and at 12-months follow-up. White and black bars represent no use of OCS and use of OCS, respectively. The difference was compared using Chi-square test. *p* < 0.05 *. Abbreviations: OCS, oral corticosteroid.

**Figure 3:**
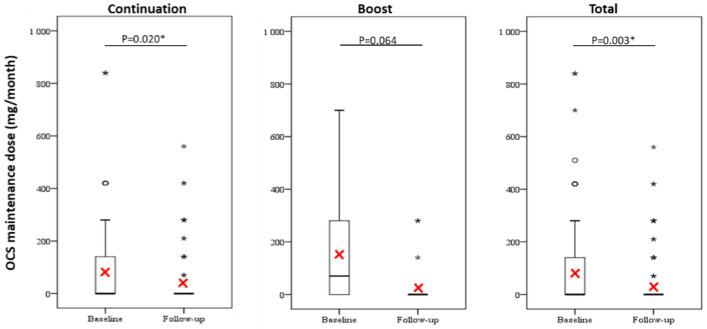
Comparison of monthly OCS maintenance doses between baseline and. 12-month follow-up. Red cross represents the mean value. The difference was compared using a paired sample *t*-test or Wilcoxon signed-rank test. *p* < 0.05 *. Abbreviations: OCS, oral corticosteroid.

**Figure 4:**
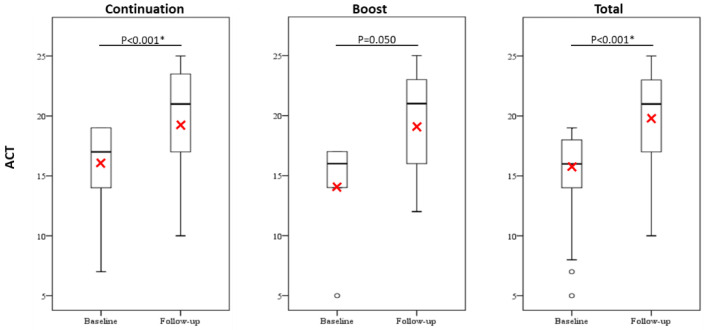
A comparison of ACT scores between baseline and the 12-month follow-up. Red cross represents the mean value. The difference was compared using the cohort whose data were available both at baseline and at the 12-month follow-up by paired sample t-test or Wilcoxon signed-rank test. *p* < 0.05 *. Abbreviations: ACT, asthma control test.

Omalizumab treatment showed a significant reduction in the frequency of OCS use as controllers (Figure 2) and the monthly OCS maintenance dose (Figure 3), and significant improvements in asthma control as determined by ACT scores (Figure 4) between the baseline and the 12-month follow-up in the total group and the continuation group but not the boost group. Furthermore, more than half of all patients achieved the minimal clinically important difference in ACT score increments of ≥3 points in all study groups (Table S1).

**Figure 5:**
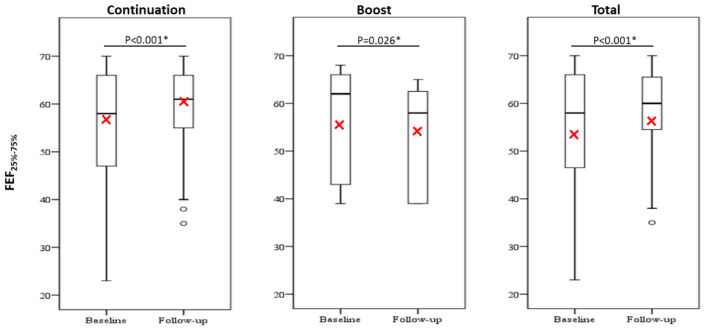
Comparison of FEF_25–75%_ between baseline and 12-months follow-up. Red cross represents the mean value. The difference was compared using the cohort whose data were available both at baseline and at 12-months follow-up by paired sample t-test or Wilcoxon signed-rank test. *p* < 0.05 *. Abbreviations: FEF_25–75%_, forced expiratory flow at 25–75%.

There were no significant changes in the inhaled maintenance medications and the pulmonary functions between the baseline and 12-month follow-up in all study groups (Figures S2−S7) except for small airway function impairment as defined by a reduction in the forced expiratory flow at 25–75% (FEF_25–75%_) (Figure 5). This shows that the continuation group and total group had a significantly increased FEF_25–75%_ at one-year follow-up when compared to the baseline. In contrast, the boost group had a significantly reduced FEF_25–75%_ between the 12-month follow-up and baseline. Detailed results are shown in Tables S1 and S2.

## 4. Discussion

To the best of our knowledge, this study is the first to compare the long-term effectiveness between continuous use and four-month boost of Omalizumab in adult patients with SAA. In contrast to the reduction in exacerbations and the improvement in small airway function with continuous Omalizumab treatment, four-month boosting Omalizumab therapy increased the exacerbation frequency and impaired small airway ventilatory function in such population. Furthermore, continuous Omalizumab therapy, rather than four-month boost of Omalizumab, significantly decreased the frequency and monthly dose of OCS as controllers and had significant improvements in asthma control. All these results were found on the basis of no significant changes in the inhaled maintenance pharmacological therapy between the baseline and the 12-month follow-up.

Contradictory to our findings that four-month boost of Omalizumab for adult SAA patients with the mean and median ages of 68.0 years and 68.0 years, respectively, significantly increased exacerbations and worsened small airway function at the 12-month follow-up, one previous study reported by Teach SJ et al. showed that four-month intervention treatment with Omalizumab initiated four to six weeks before return to school for inner-city children with allergic asthma could reduce fall exacerbations, particularly among patients with a recent exacerbation [16]. Moreover, Nopp A et al. and Vennera MDC et al. found that, after six-year Omalizumab therapy for SAA, most of the patients with a relatively younger age (median age, 50 years; range, 39–73 by Nopp A et al.; mean ± SD age, 55.7 ± 11.2 years by Vennera MDC et al.) still had clinically stable asthma one year after withdrawal from Omalizumab [17,18]. This discrepancy between our study and those reported by Teach SJ et al. Nopp A et al. and Vennera MDC et al. may mainly arise from different study subjects enrolled (adult patients with SAA in the present study versus pediatric patients with allergic asthma irrespective of disease severity in the study published by Teach SJ et al.; an older adult population in our study versus a younger adult population in the studies reported by Nopp A et al. and Vennera MDC et al.) and different treatment duration of Omalizumab (four months in the present study versus six years in the studies published by Nopp A et al. and Vennera MDC et al.) [16,17,18]. On the other hand, Domingo C et al. devised a step-down protocol for Omalizumab in SAA patients who were OCS dependent and treated with Omalizamab for at least 18 months [19]. Briefly, this study implemented the strategy by reducing half of the Omalizumab dose after 6 months of clinical stability. The protocol could not be carried out in 15 out of 35 patients (42.9%) while eight (22.9%) and 12 (34.3%) patients were partially tolerant and tolerant to withdraw, respectively. Meanwhile, all the participants had no severe exacerbations and those who were partially tolerant and tolerant successfully reduced the OCS dose in the follow-up period ranging from 12 months to 30 months. Taken together, these findings may help clarify patient selection for short-term boost of Omalizumab, and optimal treatment duration prior to and proper patient characteristic and protocol for withdrawal from Omalizumab treatment for patients with allergic asthma.

Consistent with our findings that continuous use of Omalizumab could benefit adult patients with SAA in terms of exacerbation frequency, frequency and dosage of OCS as controllers, and asthma control, previous real-world studies have shown that long-term Omalizumab therapy could substantially improve the frequency of exacerbations, GINA classifications and control and health care utilization [4,5,6,7,8,9,10,11,12,13,14]. Particularly, we found that OCS maintenance doses decreased from 85.9 ± 180.8 mg/month to 45.8 ± 106.6 mg/month and withdrew in 14 out of 36 adult SAA patients in the continuation group. Together with the findings that OCSs were significantly reduced from 7.19 ± 11.1 mg/day to 3.29 ± 11.03 mg/day and withdrawn in 74.2% of OCS-dependent IgE-mediated asthmatic patients with a follow-up period of 17.2 ± 8.5 months (range: 4–34) [20], this indicates that long-term, continuous Omalizumab treatment could substantially decrease the requirement of OCS as controller in SAA patients. Interestingly, we also found that continuous Omalizumab treatment did not improve lung function except for small airway ventilation as determined by FEF_25–75%_, which was similar to those published by Domingo C et al. who found that pulmonary functions did not change significantly following long-term use of Omalizumab [20], although reports that Omalizumab therapy could improve forced expiratory volume in one second (FEV1) by around 12% in patients with SAA have been well documented [7,21]. These differences may be due to the age of the participants. A younger study population was enrolled in previous studies by Brusselle G et al. and Barnes N et al. (mean ± SD age, 48.17 ± 17.18 and 41.26 ± 14.52 years, respectively), whereas the patients in our study and those in the study reported by Domingo C et al. were notably older (mean ± SD age, 59.9 ± 15.6 years and 50.8 ± 17.0) [7,20,21].

A strength of the current study was that whether participants received continuous or boosting Omalizumab treatment during the follow-up period depended on the judgment of the SAA committee members of Taiwan NHI, making it less biased toward study group categorization and the outcomes of interest. Furthermore, this real-life study was conducted by qualified pulmonologists and immunologists who were actively involved in SAA management in a referral hospital setting and confirmed the diagnosis of SAA based on the GINA recommendations, elevated serum total IgE level and the presence of a history of allergic reaction, tested allergens and/or concomitant atopic diseases, ensuring a valid study population of patients with SAA although in-vivo skin prick test for determination of allergy had not been performed for the participants [1]. This compensates for several important limitations of the present study, including that underestimation of exacerbation frequency is possible due to its retrospective design. Moreover, there is a small number of subjects categorized into the boost group.

As discussed above, in contrast to the previous studies that showed short-term Omalizumab treatment initiated before return to school could reduce fall exacerbations in a pediatric study population while 52-week therapy with Omalizumab improved FEV1 significantly in a younger adult study population [7,16,21], the current study found that the exacerbation frequency increased and the small airway function decreased at the 12-month follow-up when Omalizumab was boosted for four months while lung functions did not improve after 12-month continuous Omalizumab treatment in an older adult study population. These findings imply that the younger age of the patients may be a predictor for tolerating short-term boost of Omalizumab therapy and for pulmonary function improvement when treating with long-term Omalizumab in patients with allergic asthma although these should be validated in the future. Moreover, further studies should be conducted to determine the optimal strategy for the use of Omalizumab in patients with allergic asthma.

## 5. Conclusions

In contrast to the long-term effectiveness of continuous Omalizumab treatment, boost of Omalizumab treatment for four months leads to worse clinical outcomes at the 12-month follow-up period in adult patients with SAA. These findings support previous reports and confirm the benefits of persistent Omalizumab treatment in adult patients with SAA, although the optimal strategy for the use of Omalizumab remain unknown.

## Figures and Tables

**Table 1:** Baseline information for the enrolled participants.

	Continuation (*n* = 110)	Boost (*n* = 14)	Total (*n* = 124)
**Age (years)**			
Mean ± SD	59.9 ± 15.6	68.0 ± 15.7	60.8 ± 15.7
Median (Q1, Q3)	62.0 (48.8, 70.0)	68.0 (55.8, 82.3)	62.0 (50.0, 71.0)
**Male gender**	58 (52.7%)	9 (64.3%)	67 (54.0%)
**Height (cm)**			
Mean ± SD	175.6 ± 133.7	159.7 ± 8.8	173.8 ± 126.0
Median (Q1, Q3)	163.0 (156.0, 169.8)	163.0 (151.3, 168.1)	163.0 (155.6, 169.2)
**Weight (kg)**			
Mean ± SD	69.0 ± 12.3	71.0 ± 16.1	69.2 ± 12.7
Median (Q1, Q3)	67.8 (60.5, 77.0)	63.5 (58.5, 87.8)	67.3 (60.4, 77.0)
**Smoking (pack-year)**			
Mean ± SD	9.4 ± 19.6	8.6 ± 13.6	9.3 ± 19.0
Median (Q1, Q3)	0.0 (0.0, 10.0)	0.0 (0.0, 20.0)	0.0 (0.0, 13.8)
**Smoking history**			
Never smoker	73 (66.4%)	9 (64.3%)	82 (66.1%)
Ex-smoker	31 (28.2%)	5 (35.7%)	36 (29.0%)
Current smoker	6 (5.5%)	0 (0.0%)	6 (4.8%)
**Time for asthma treatment history (years)**			
Mean ± SD	3.8 ± 3.6	2.9 ± 2.7	3.7 ± 3.5
Median (Q1, Q3)	2.9 (0.8, 6.3)	2.1 (1.2, 3.8)	2.8 (0.9, 5.8)
**Total IgE (kU/L)**			
Mean ± SD	743.0 ± 736.6	747.8 ± 691.8	743.5 ± 729.0
Median (Q1, Q3)	507.5 (280.8, 958.8)	483.5 (193.8, 1530.8)	507.5 (274.8, 968.3)
**WBC (10^9^/L)**			
Mean ± SD	8.5 ± 2.9	8.4 ± 3.5	8.5 ± 2.9
Median (Q1, Q3)	7.9 (6.5, 9.7)	7.1 (6.1, 11.2)	7.9 (6.5, 9.8)
**Blood eosinophil percentage (%)**			
Mean ± SD	4.3 ± 4.1	6.7 ± 12.6	4.5 ± 5.7
Median (Q1, Q3)	2.9 (1.6, 5.5)	1.5 (1.2, 7.0)	2.9 (1.4, 5.6)
**Blood absolute eosinophil count (cells/μL)**			
Mean ± SD	348.0 ± 434.9	827.2 ± 2136.3	402.1 ± 820.5
Median (Q1, Q3)	225.8 (119.2, 418.7)	124.3 (63.2, 496.2)	223.2 (111.3, 422.2)
**Number of allergens tested**			
Mean ± SD	2.0 ± 1.5	1.3 ± 1.3	1.9 ± 1.5
Median (Q1, Q3)	2.0 (1.0, 3.0)	1.0 (0.0, 2.3)	2.0 (1.0, 3.0)
0	23 (20.9%)	4 (28.6%)	27 (21.8%)
1	22 (20.0%)	6 (42.9%)	28 (22.6%)
2	26 (23.6%)	1 (7.1%)	27 (21.8%)
3	22 (20.0%)	2 (14.3%)	24 (19.4%)
4	11 (10.0%)	1(7.1%)	12 (9.7%)
5	4 (3.6%)	0 (0.0%)	4 (3.2%)
6	1 (0.9%)	0 (0.0%)	1 (0.8%)
7	1 (0.9%)	0 (0.0%)	1 (0.8%)
**Omalizumab dose (mg/month)**			
Mean ± SD	451.6 ± 230.1	540.0 ± 171.0	455.4 ± 228.0
Median (Q1, Q3)	450.0 (300.0, 600.0)	600.0 (375.0, 675.0)	450.0 (300.0, 600.0)
**Oral maintenance medication except for OCS**			
None	10 (9.1%)	1 (7.1%)	11 (8.9%)
Montelukast alone	65 (59.1%)	8 (57.1%)	73 (58.9%)
Methylxanthines alone	11 (10.0%)	2 (14.3%)	13 (10.5%)
Montelukast + Methylxanthines	24 (21.8%)	3 (21.4%)	27 (21.8%)
**Co-morbidity**			
Depression	21 (19.1%)	2 (14.3%)	23 (18.5%)
Insomnia	24 (21.8%)	2 (14.3%)	26 (21.0%)
Osteoporosis	12 (10.9%)	0 (0.0%)	12 (9.7%)
Cerebrovascular disease	11 (10.0%)	3 (21.4%)	14 (11.3%)
GERD	31 (28.2%)	6 (42.9%)	37 (29.8%)
COPD	27 (24.5%)	9 (64.3%)	36 (29.0%)
DM	20 (18.2%)	3 (21.4%)	23 (18.5%)
Allergic rhinitis	92 (83.6%)	10 (71.4%)	102 (82.3%)
Atopic dermatitis	11 (10.0%)	0 (0.0%)	11 (8.9%)
Urticaria	33 (30.0%)	5 (35.7%)	38 (30.6%)
Allergic Conjunctivitis	17 (15.5%)	1 (7.1%)	18 (14.5%)
Food or drug allergy	10 (9.1%)	0 (0.0%)	10 (8.1%)
AERD	1 (0.9%)	0 (0.0%)	1 (0.8%)
OSAS	5 (4.5%)	0 (0.0%)	5 (4.0%)

Abbreviations: AERD, aspirin-exacerbated respiratory disease; COPD, chronic obstructive pulmonary disease; DM, diabetes mellitus; GERD, gastro-esophageal reflux disease; IgE, immunoglobulin E; OCS, oral corticosteroid; OSAS, obstructive sleep apnea syndrome; Q, quartile; SD, standard deviation; WBC, white blood count.

## Data Availability

Data supporting reported results can be found at Lab. 114 in Taichung Veterans General Hospital, Taichung, Taiwan.

## Supplementary Materials

The following are available online at https://www.mdpi.com/2077-0383/10/4/707/s1, Table S1: Changes in the asthma control, frequency of oral corticosteroid use as controllers and inhaled maintenance pharmacological therapies, monthly oral corticosteroid maintenance dose, and exacerbation frequency after Omalizumab treatment; Table S2: Changes in the lung function after Omalizumab treatment; Figure S1: The patient enrollment flow chart; Figure S2: The comparison of inhaled maintenance pharmacological therapies between baseline and one-year follow-up. White and black bars represented medium-dose ICS/LABA ± Tiotropium and high-dose ICS/LABA ± Tiotropium, respectively. The difference was compared using Chi-square test at a significant difference of *p*-value < 0.05. Abbreviations: ICS, inhaled corticosteroid; LABA, long-acting beta-agonist; Figure S3: The FEV1/FVC at baseline and at 12-month follow-up. Red cross represented the mean value. The difference was compared using the cohort whose data were available both at baseline and at 12-month follow-up by paired sample *t*-test or Wilcoxon signed-rank test at a significant difference of *p*-value < 0.05. Abbreviations: FEV1, forced expiratory volume in one second; FVC, forced vital capacity; Figure S4: The comparison of FEV1 between baseline and 12-month follow-up. Red cross represented the mean value. The difference was compared using the cohort whose data were available both at baseline and at 12-month follow-up by paired sample *t*-test or Wilcoxon signed-rank test at a significant difference of *p*-value < 0.05. Abbreviations: FEV1, forced expiratory volume in one second; Figure S5: The FEV1 % predicted at baseline and at one-year follow-up. Red cross represented the mean value. The difference was compared using the cohort whose data were available both at baseline and at 12-month follow-up by paired sample *t*-test or Wilcoxon signed-rank test at a significant difference of *p*-value < 0.05. Abbreviations: FEV1, forced expiratory volume in one second; Figure S6: The comparison of FVC between baseline and 12-month follow-up. Red cross represented the mean value. The difference was compared using the cohort whose data were available both at baseline and at 12-month follow-up by paired sample t-test or Wilcoxon signed-rank test at a significant difference of *p*-value < 0.05. Abbreviations: FVC, forced vital capacity; Figure S7: The comparison of FVC % predicted between baseline and one-year follow-up. Red cross represented the mean value. The difference was compared using the cohort whose data were available both at baseline and at 12-month follow-up by paired sample t-test or Wilcoxon signed-rank test at a significant difference of *p*-value < 0.05. Abbreviations: FVC, forced vital capacity.
