# Supplementary material for: The Long-Term Effectiveness of Omalizumab in Adult Patients with Severe Allergic Asthma: Continuous Treatment Versus Boosting Treatment
Source: J Clin Med. 2021 Feb 11;10(4):707. doi: 10.3390/jcm10040707 (PMC7916868; doi:10.3390/jcm10040707)
Supplement: Supplementary file 1 [file jcm-10-00707-s001.pdf]

Table S1. Changes in the asthma control, frequency of oral corticosteroid use as controllers and inhaled maintenance pharmacological therapies, monthly oral corticosteroid maintenance dose, and exacerbation frequency after Omalizumab treatment.

	Baseline	Follow-Up	Change from Baseline
**Continuation (*n* = 110)**			
**Number of exacerbation**			
Number of patients evaluated	110 (100%)	110 (100%)	110 (100%)
Mean ± SD	0.8 ± 1.5	0.5 ± 1.0	−0.3 ± 1.4
Median (Q1, Q3)	0.0 (0.0, 1.0)	0.0 (0.0, 1.0)	0.0 (0.0, 0.0)
Persistent presence	NA	NA	14 (12.7%)
Complete prevention	NA	NA	18 (16.4%)
Occurrence from nil	NA	NA	17 (15.5%)
Never present	NA	NA	61 (55.5%)
**Use of OCS as controller**	36 (32.7%)	22 (20.0%)	−14 (−12.7%)
**OCS maintenance dose (mg per month)** †			
Number of patients evaluated	110 (100%)	110 (100%)	110 (100%)
Mean ± SD	85.9 ± 180.8	45.8 ± 106.6	−40.1 ± 178.3
Median (Q1, Q3)	0.0 (0.0, 140.0)	0.0 (0.0, 0.0)	0.0 (0.0, 0.0)
**ACT**			
Number of patients evaluated	81 (73.6%)	76 (69.1%)	76 (69.1%)
Mean ± SD	15.9 ± 3.1	19.8 ± 4.4	3.8 ± 4.6
Median (Q1, Q3)	17.0 (14.0, 19.0)	21.0 (17.0, 23.8)	4.0 (1.0, 7.0)
≧3 (Responder)	NA	NA	47 (61.8%)
<3 (Non-responder)	NA	NA	29 (38.2%)
**Inhaled maintenance pharmacological therapy**			
Medium-dose ICS/LABA +/- Tiotropium	46 (41.8%)	52 (47.3%)	6 (4.8%)
High-dose ICS/LABA +/- Tiotropium	64 (58.2%)	58 (52.7%)	−6 (−4.8%)
**Boost (*n* = 14)**			
**Number of exacerbation**			
Number of patients evaluated	14 (100%)	14 (100%)	14 (100%)
Mean ± SD	0.7 ± 1.4	2.9 ± 3.6	2.1 ± 3.7
Median (Q1, Q3)	0.0 (0.0, 0.8)	2.0 (0.0, 4.0)	2.0 (0.0, 4.0)
Persistent presence	NA	NA	2 (14.3%)
Complete prevention	NA	NA	1 (7.1%)
Occurrence from nil	NA	NA	7 (50.0%)
Never present	NA	NA	4 (28.6%)
**Use of OCS as controller**	7 (50.0%)	3 (21.4%)	−4 (−28.6%)
**OCS maintenance dose (mg per month)**†			
Number of patients evaluated	14 (100%)	14 (100%)	14 (100%)
Mean ± SD	171.4 ± 221.5	50.0 ± 104.3	−121.4 ± 219.6
Median (Q1, Q3)	70.0 (0.0, 280.0)	0.0 (0.0, 35.0)	−70.0 (−280.0, 0.0)
**ACT**			
Number of patients evaluated	12 (85.7%)	9 (64.3%)	9 (64.3%)
Mean ± SD	13.9 ± 3.9	19.7 ± 4.7	5.1 ± 5.4
Median (Q1, Q3)	15.0 (14.0, 16.8)	21.0 (15.0, 23.5)	7.0 (−1.0, 8.0)
≧3 (Responder)	NA	NA	6 (66.7%)
<3 (Non-responder)	NA	NA	3 (33.3%)
**Inhaled maintenance pharmacological therapy**			
Medium-dose ICS/LABA +/- Tiotropium	7 (50.0%)	8 (57.1%)	1 (0.8%)
High-dose ICS/LABA +/- Tiotropium	7 (50.0%)	6 (42.9%)	−1 (−0.8%)
**Total (*n* = 124)**			
**Number of exacerbation**			

Number of patients evaluated	124 (100%)	124 (100%)	124 (100%)
Mean ± SD	0.8 ± 1.5	0.8 ± 1.7	0.0 ± 1.9
Median (Q1, Q3)	0.0 (0.0, 1.0)	0.0 (0.0, 1.0)	0.0 (0.0, 0.0)
Persistent presence	NA	NA	16 (12.9%)
Complete prevention	NA	NA	19 (15.3%)
Occurrence from nil	NA	NA	24 (19.4%)
Never present	NA	NA	65 (52.4%)
**Use of OCS as controller**	43 (34.7%)	25 (20.2%)	−18 (−14.5%)
**OCS maintenance dose (mg per month)** †			
Number of patients evaluated	124 (100%)	124 (100%)	124 (100%)
Mean ± SD	95.6 ± 186.8	46.3 ± 105.9	−49.3 ± 184.2
Median (Q1, Q3)	0.0 (0.0, 140.0)	0.0 (0.0, 0.0)	0.0 (−96.3, 0.0)
**ACT**			
Number of patients evaluated	93 (75.0%)	85 (68.5%)	85 (68.5%)
Mean ± SD	15.6 ± 3.3	18.8 ± 4.4	4.0 ± 4.7
Median (Q1, Q3)	16.0 (14.0, 18.0)	21.0 (17.0, 23.5)	4.0 (0.5, 7.0)
≧3 (Responder)	NA	NA	53 (62.4%)
<3 (Non-responder)	NA	NA	32 (37.6%)
**Inhaled maintenance pharmacological therapy**			
Medium-dose ICS/LABA +/- Tiotropium	53 (42.7%)	60 (48.4%)	7 (5.6%)
High-dose ICS/LABA +/- Tiotropium	71 (57.3%)	64 (51.6%)	−7 (−5.6%)

†The steroid dose was conversed to equivalent dose of prednisone. Abbreviations: ACT, asthma control test; ICS, inhaled corticosteroid; LABA, long-acting beta-agonist; NA, not applicable; OCS, oral corticosteroid; Q, quartile; SD, standard deviation.

Table S2. Changes in the lung function after Omalizumab treatment.

	Baseline	Follow-Up	Change from Baseline
**Continuation (*n* = 110)**			
**Pre-BT FEV1/FVC (%)**			
Number of patients evaluated	85 (77.3%)	84 (76.4%)	68 (61.8%)
Mean ± SD	64.1 ± 14.2	63.8 ± 13.9	−1.0 ± 7.8
Median (Q1, Q3)	64.0 (55.0, 73.0)	64.0 (53.0, 72.0)	−0.5 (−5.0, 4.8)
**Pre-BT FEV1 (L)**			
Number of patients evaluated	85 (77.3%)	84 (76.4%)	68 (61.8%)
Mean ± SD	1.8 ± 0.8	1.9 ± 0.8	0.0 ± 0.3
Median (Q1, Q3)	1.7 (1.1, 2.3)	1.7 (1.2, 2.4)	0.0 (−0.2, 0.2)
**Pre-BT FEV1 % predicted**			
Number of patients evaluated	85 (77.3%)	84 (76.4%)	68 (61.8%)
Mean ± SD	65.8 ± 21.7	69.4 ± 21.9	1.3 ± 11.3
Median (Q1, Q3)	64.0 (47.5, 80.5)	71.5 (52.3, 83.8)	0.0 (−7.8, 9.0)
**Pre-BT FVC (L)**			
Number of patients evaluated	85 (77.3%)	84 (76.4%)	68 (61.8%)
Mean ± SD	2.7 ± 0.9	2.9 ± 1.0	0.1 ± 0.4
Median (Q1, Q3)	2.7 (2.0, 3.3)	2.8 (2.2, 3.5)	0.0 (−0.2, 0.3)
**Pre-BT FVC % predicted**			
Number of patients evaluated	85 (77.3%)	84 (76.4%)	68 (61.8%)
Mean ± SD	84.2 ± 20.9	88.1 ± 19.4	2.4 ± 12.5
Median (Q1, Q3)	84.0 (68.5, 97.5)	89.0 (76.8, 100.0)	1.5 (−5.8, 9.5)
**Pre-BT $FEF_{25–75}$ (%)**			
Number of patients evaluated	85 (77.3%)	84 (76.4%)	68 (61.8%)
Mean ± SD	54.2 ± 11.6	58.9 ± 9.1	4.3 ± 5.3
Median (Q1, Q3)	56.0 (45.5, 66.0)	60.0 (54.0, 66.0)	4.0 (0.0, 6.8)

**Boost (*n* = 14)**			
**Pre-BT FEV1/FVC (%)**			
Number of patients evaluated	12 (85.7%)	9 (64.3%)	7 (50.0%)
Mean ± SD	63.1 ± 14.2	61.3 ± 10.9	−1.4 ± 11.0
Median (Q1, Q3)	68.5 (46.8, 75.0)	57.0 (54.0, 69.5)	−2.0 (−13.0, 8.0)
**Pre-BT FEV1 (L)**			
Number of patients evaluated	12 (85.7%)	9 (64.3%)	7 (50.0%)
Mean ± SD	1.6 ± 0.7	1.7 ± 0.5	0.1 ± 0.4
Median (Q1, Q3)	1.6 (1.1, 2.1)	1.6 (1.2, 2.0)	−0.1 (−0.2, 0.6)
**Pre-BT FEV1 % predicted**			
Number of patients evaluated	12 (85.7%)	9 (64.3%)	7 (50.0%)
Mean ± SD	67.3 ± 27.8	72.8 ± 25.7	2.1 ± 18.3
Median (Q1, Q3)	67.5 (44.8, 74.0)	64.0 (55.5, 91.5)	−6.0 (−7.0, 23.0)
**Pre-BT FVC (L)**			
Number of patients evaluated	12 (85.7%)	9 (64.3%)	7 (50.0%)
Mean ± SD	2.5 ± 0.8	2.7 ± 0.9	0.3 ± 0.5
Median (Q1, Q3)	2.6 (1.7, 3.1)	2.7 (2.0, 3.5)	0.2 (−0.3, 0.8)
**Pre-BT FVC % predicted**			
Number of patients evaluated	12 (85.7%)	9 (64.3%)	7 (50.0%)
Mean ± SD	86.0 ± 33.8	96.6 ± 39.1	7.0 ± 17.4
Median (Q1, Q3)	75.5 (68.8, 84.0)	85.0 (69.0, 110.5)	8.0 (−7.0, 22.0)
**Pre-BT $FEF_{25–75}$ (%)**			
Number of patients evaluated	12 (85.7%)	9 (64.3%)	7 (50.0%)
Mean ± SD	57.3 ± 12.2	53.3 ± 13.8	−3.1 ± 1.7
Median (Q1, Q3)	63.5 (43.8, 67.8)	58.0 (39.0, 64.0)	−4.0 (−4.0, −2.0)
**Total (*n* = 124)**			
**Pre-BT FEV1/FVC (%)**			
Number of patients evaluated	97 (78.2%)	93 (75.0%)	75 (60.5%)
Mean ± SD	64.0 ± 14.2	63.6 ± 13.5	−1.0 ± 8.0
Median (Q1, Q3)	65.0 (55.0, 74.5)	63.0 (53.0, 72.0)	−1.0 (−5.0, 5.0)
**Pre-BT FEV1 (L)**			
Number of patients evaluated	97 (78.2%)	93 (75.0%)	75 (60.5%)
Mean ± SD	1.7 ± 0.7	1.8 ± 0.8	0.0 ± 0.3
Median (Q1, Q3)	1.7 (1.1, 2.3)	1.7 (1.3, 2.4)	0.0 (−0.2, 0.2)
**Pre-BT FEV1 % predicted**			
Number of patients evaluated	97 (78.2%)	93 (75.0%)	75 (60.5%)
Mean ± SD	66.0 ± 22.4	69.7 ± 21.8	1.4 ± 11.9
Median (Q1, Q3)	66.0 (47.5, 79.5)	69.0 (52.5, 83.5)	0.0 (−7.0, 9.0)
**Pre-BT FVC (L)**			
Number of patients evaluated	97 (78.2%)	93 (75.0%)	75 (60.5%)
Mean ± SD	2.7 ± 0.9	2.8 ± 0.9	0.1 ± 0.4
Median (Q1, Q3)	2.7 (1.9, 3.3)	2.8 (2.2, 3.5)	0.1 (−0.2, 0.3)
**Pre-BT FVC % predicted**			
Number of patients evaluated	97 (78.2%)	93 (75.0%)	75 (60.5%)
Mean ± SD	84.5 ± 23.2	88.9 ± 22.2	2.8 ± 12.9
Median (Q1, Q3)	83.0 (68.5, 97.0)	89.0 (76.0, 100.5)	2.0 (−6.0, 12.0)
**Pre-BT $FEF_{25–75\%}$**			
Number of patients evaluated	97 (78.2%)	93 (75.0%)	75 (60.5%)
Mean ± SD	54.6 ± 11.7	58.4 ± 9.7	3.6 ± 5.5
Median (Q1, Q3)	57.0 (45.5, 66.0)	60.0 (54.0, 66.0)	4.0 (−1.0, 6.0)

Abbreviations: BT, bronchodilator test; $FEF_{25–75\%}$, forced expiratory flow at 25–75%; FEV1, forced expiratory volume in one second; FVC, forced vital capacity; also see Supplementary Table S1.

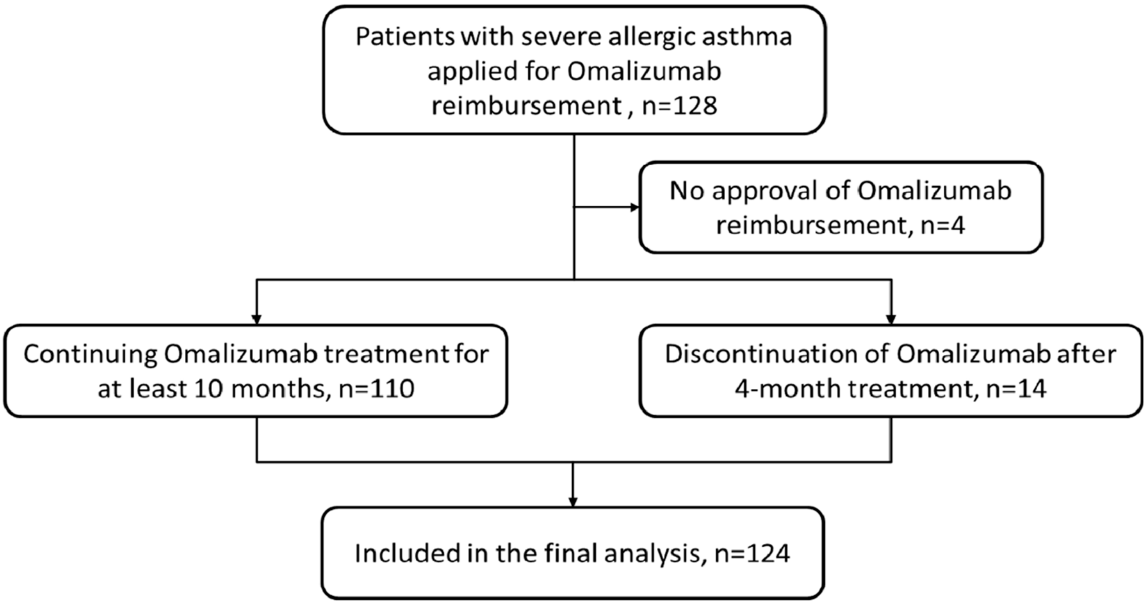

Figure S1. The patient enrollment flow chart.

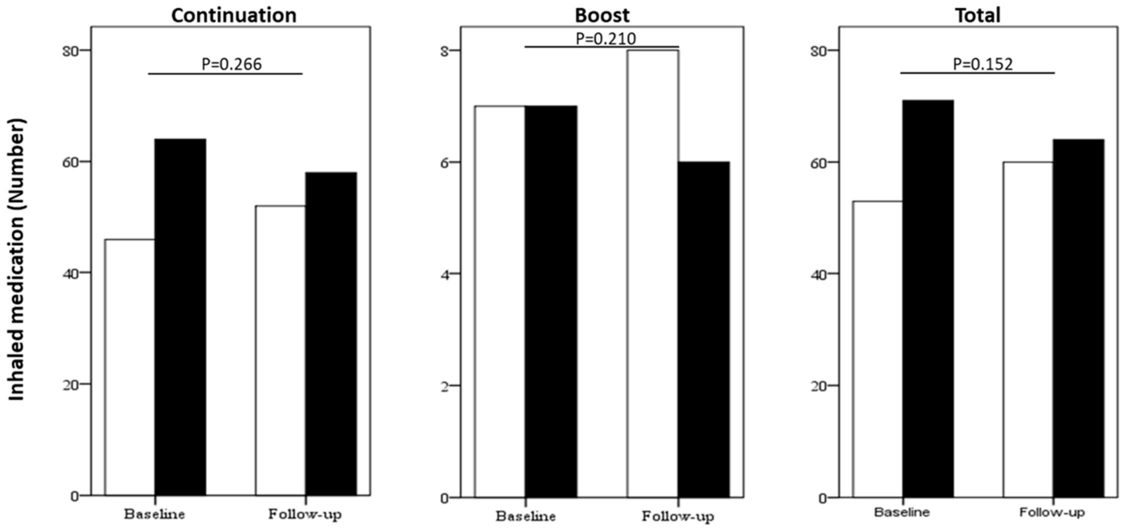

Figure S2. The comparison of inhaled maintenance pharmacological therapies between baseline and one-year follow-up. White and black bars represented medium-dose ICS/LABA +/- Tiotropium and high-dose ICS/LABA +/- Tiotropium, respectively. The difference was compared using Chi-square test at a significant difference of P-value < 0.05. Abbreviations: ICS, inhaled corticosteroid; LABA, long-acting beta-agonist.

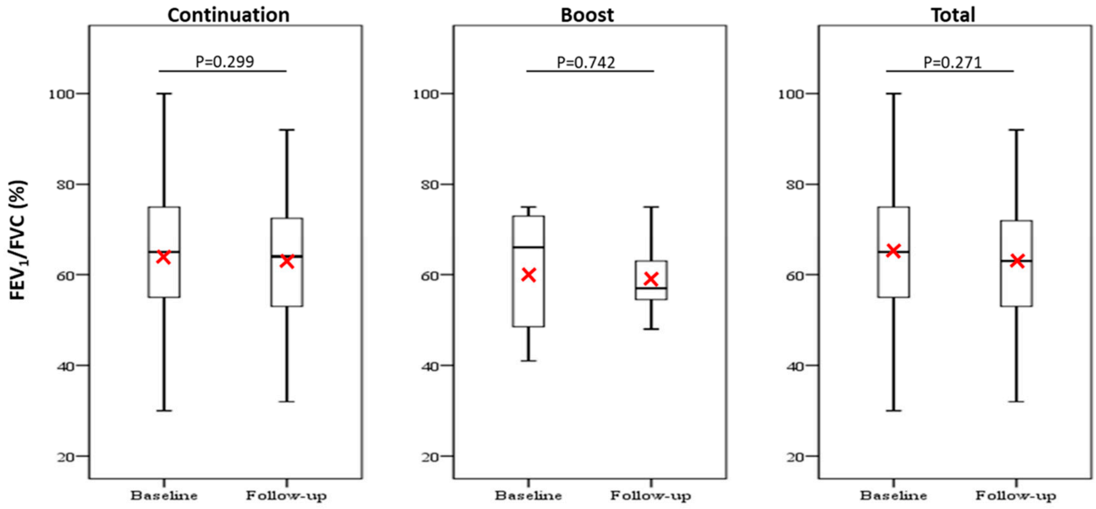

Figure S3. The FEV1/FVC at baseline and at 12-month follow-up. Red cross represented the mean value. The difference was compared using the cohort whose data were available both at baseline and at 12-month follow-up by paired sample *t*-test or Wilcoxon signed-rank test at a significant difference of *P*-value < 0.05. Abbreviations: FEV1, forced expiratory volume in one second; FVC, forced vital capacity.

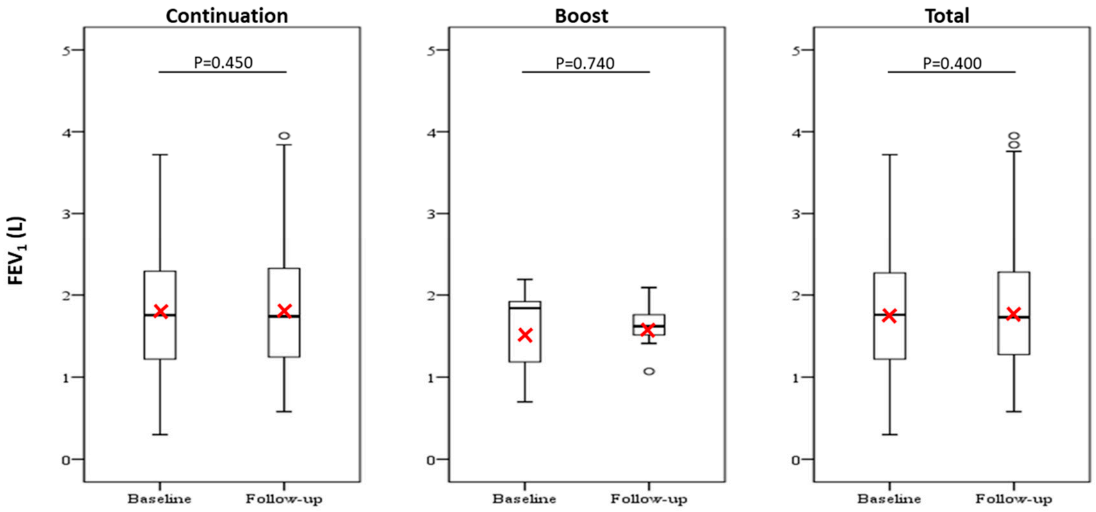

Figure S4. The comparison of FEV1 between baseline and 12-month follow-up. Red cross represented the mean value. The difference was compared using the cohort whose data were available both at baseline and at 12-month follow-up by paired sample *t*-test or Wilcoxon signed-rank test at a significant difference of *P*-value < 0.05. Abbreviations: FEV1, forced expiratory volume in one second.

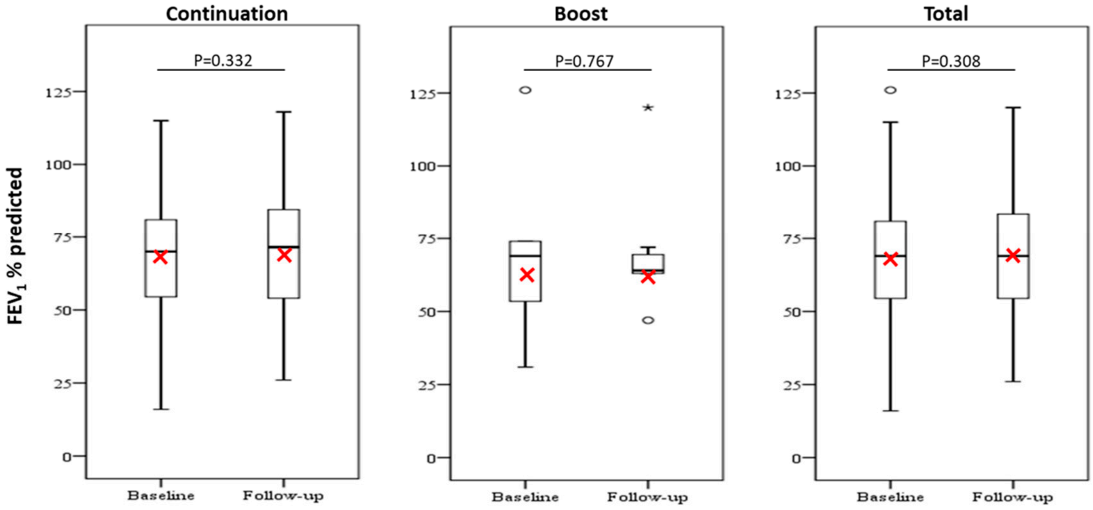

Figure S5. The FEV1 % predicted at baseline and at one-year follow-up. Red cross represented the mean value. The difference was compared using the cohort whose data were available both at baseline and at 12-month follow-up by paired sample *t*-test or Wilcoxon signed-rank test at a significant difference of *P*-value < 0.05. Abbreviations: FEV1, forced expiratory volume in one second.

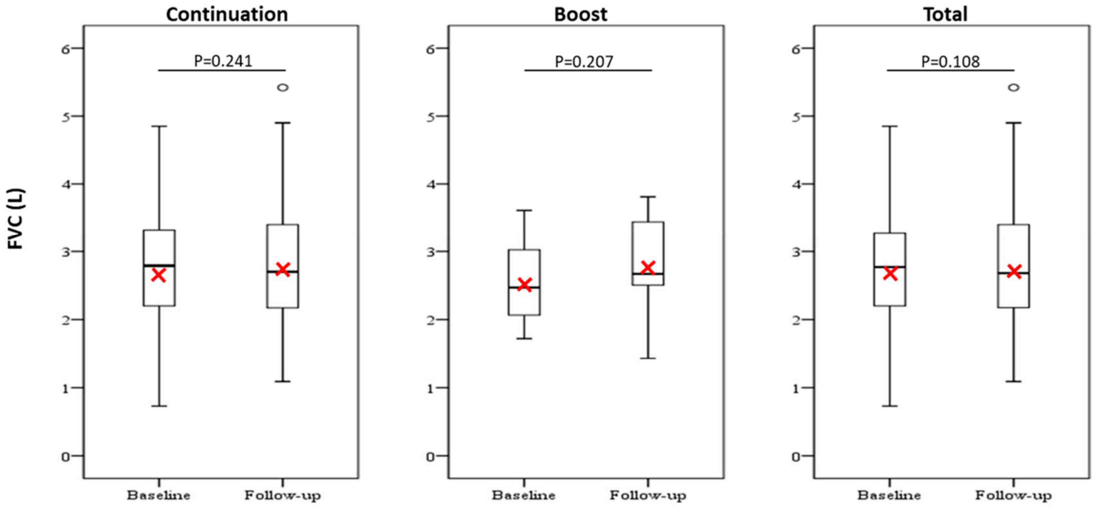

Figure S6. The comparison of FVC between baseline and 12-month follow-up. Red cross represented the mean value. The difference was compared using the cohort whose data were available both at baseline and at 12-month follow-up by paired sample t-test or Wilcoxon signed-rank test at a significant difference of *P*-value < 0.05. Abbreviations: FVC, forced vital capacity.

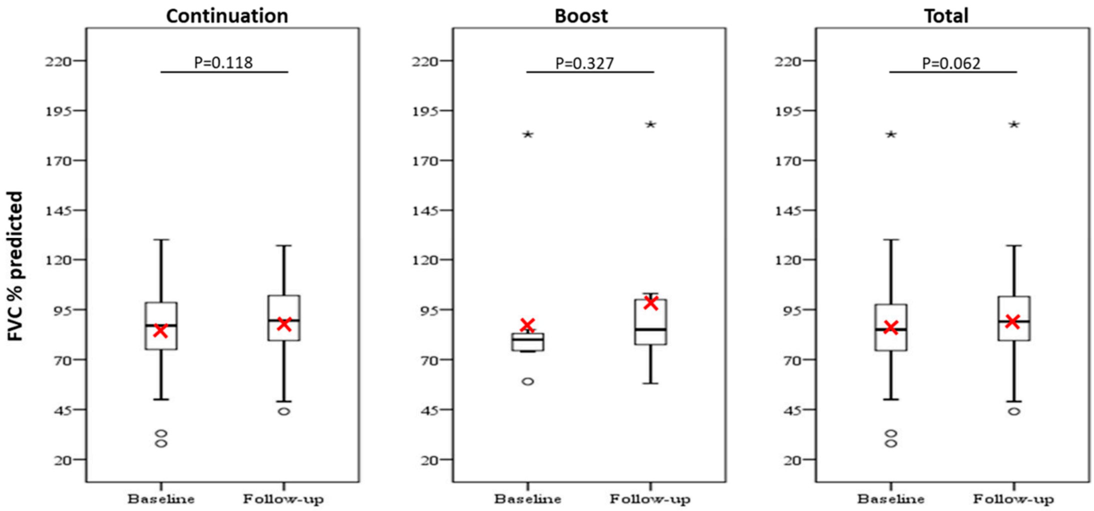

Figure S7. The comparison of FVC % predicted between baseline and one-year follow-up. Red cross represented the mean value. The difference was compared using the cohort whose data were available both at baseline and at 12-month follow-up by paired sample *t*-test or Wilcoxon signed-rank test at a significant difference of *P*-value < 0.05. Abbreviations: FVC, forced vital capacity.
